# Supplementary material for: Analysis of the Probiotic Potential of Lactiplantibacillus plantarum LB1_P46 Isolated from the Mexican Fermented Pulque Beverage: A Functional and Genomic Analysis
Source: Microorganisms. 2024 Aug 12;12(8):1652. doi: 10.3390/microorganisms12081652 (PMC11356911; doi:10.3390/microorganisms12081652)
Supplement: Supplementary file 1 [file microorganisms-12-01652-s001.zip › Figure S2.pdf]

Analysis of the potential probiotic potential of *Lactiplantibacillus plantarum* LB1_P46 isolated from the Mexican fermented pulque beverage: A functional and genomic analysis

Martha Giles-Gómez, Ximena Morales Huerta, Rodolfo Pastelín-Palacios, Constantino López-Macías, Mayrene Sarai Flores Montesinos, Fernando Astudillo-Melgar, and Adelfo Escalante

Figure S2. Antimicrobial activity of *Lactobacillus casei* Shirota as probiotic control against pathogenic bacteria. Bacterial strains used *Salmonella enterica* serovar Typhimurium ATCC14028 (Sal), *Escherichia coli* 11229 (Eco), *Pseudomonas aeruginosa* ATCC27853 (Pae), *Listeria monocytogenes* CFQ-B-103 (Lmo), *Streptococcus pyogenes* CFQ-B-128 (Spy), *Staphylococcus aureus* ATCC6538 (Sau), *Enterococcus faecalis* CFQ-B- (Efa), and *B. cereus* CFQ-B-230 (Bce).

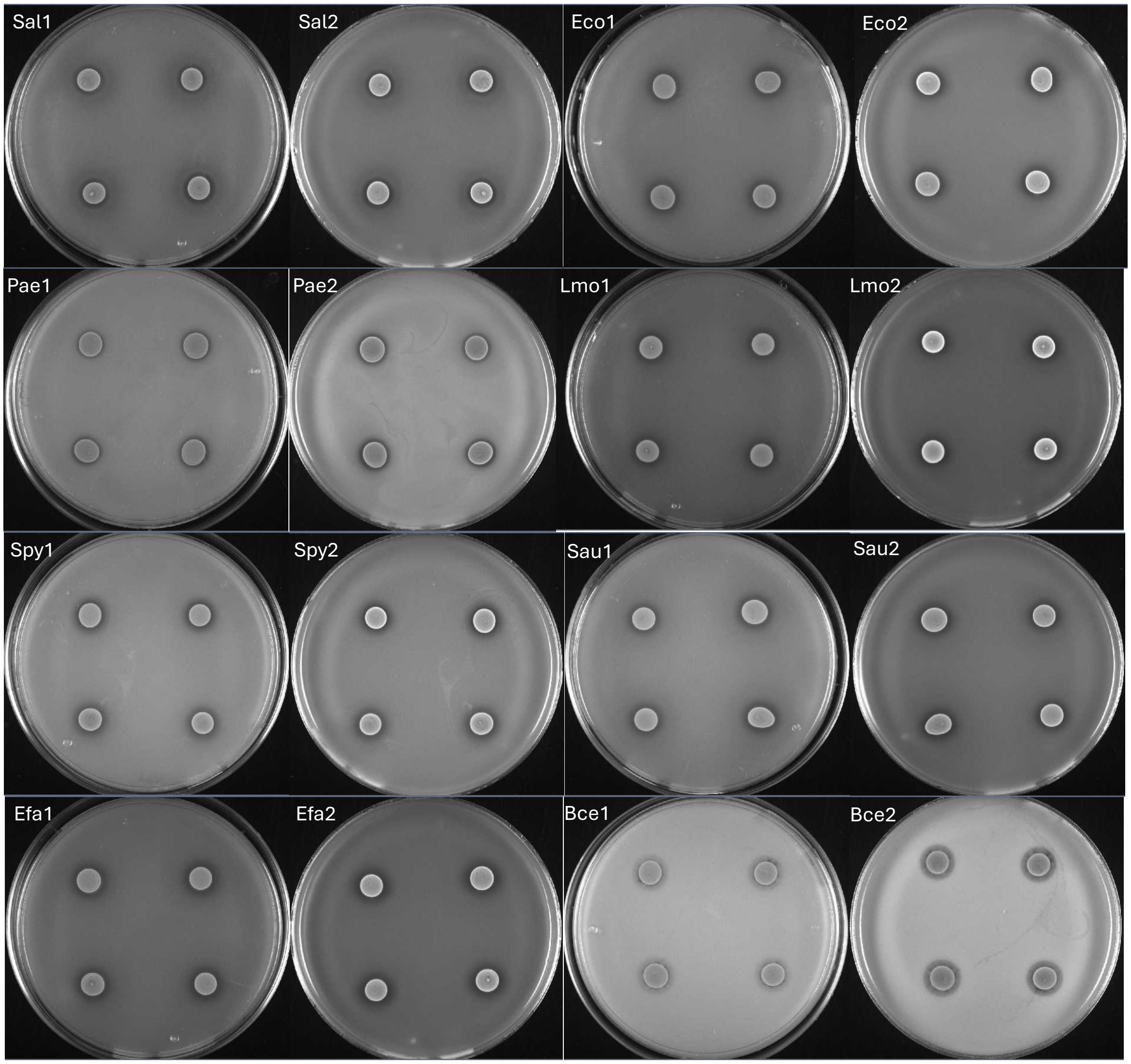
Sal1
Sal2
Eco1
Eco2
Pae1
Pae2
Lmo1
Lmo2
Spy1
Spy2
Sau1
Sau2
Efa1
Efa2
Bce1
Bce2
